# Metabotropic glutamate receptor 5 deficiency inhibits neutrophil infiltration after traumatic brain injury in mice

**DOI:** 10.1038/s41598-017-10201-8

**Published:** 2017-08-30

**Authors:** Ting Yang, Yang-Wuyue Liu, Li Zhao, Hao Wang, Nan Yang, Shuang-Shuang Dai, Fengtian He

**Affiliations:** 1 Department of Biochemistry and Molecular Biology, Third Military Medical University, Chongqing, 400038 PR China; 2 Department of Neurosurgery, Research Institute of Surgery and Daping Hospital, Third Military Medical University, Chongqing, 400042 PR China; 3 Molecular Biology Center, State Key Laboratory of Trauma, Burn, and Combined Injury, Daping Hospital, Third Military Medical University, Chongqing, 400042 PR China

## Abstract

Both brain native inflammatory cells and infiltrated peripheral white blood cells (WBCs) are primary participants in the brain inflammatory damage post-TBI. Metabotropic glutamate receptor 5 (mGluR5) has been reported to regulate microglias and astrocytes to affect inflammation after TBI, but its effect on modulating infiltrated peripheral WBCs remains unclear. In a mouse moderate TBI model, we found that mGluR5 knockout (KO) significantly reduced neutrophil infiltration and inflammatory cytokine expression in the brain at 24 hours post TBI, which was accompanied by improved neurological dysfunction. Further investigation indicated that mGluR5 KO reduced the permeability of blood-brain barrier (BBB), the entrance for neutrophils to enter brain, and markedly decreased the mRNA levels of neutrophil-associated chemokines in brain tissue, including CXCL1, CXCL2, CCL2, CCL4 and CCL5. Using brain microvascular endothelial cells (BMECs), neutrophils and a BBB model *in vitro*, we confirmed the inhibitory effect of mGluR5 deficiency on neutrophil infiltration and demonstrated that blockade of protein kinase C (PKC) signaling was involved in it. These results provide insight into the role of mGluR5 in the regulation of inflammation in the acute phase of TBI, which may provide novel clues for TBI therapy.

## Introduction

Traumatic brain injury (TBI) is a common and often life-threatening clinical condition. Approximately 30–35% of patients with moderate or severe TBI die within the first 30 days after hospital admission, whereas survivors suffer from motor and neuropsychological malfunctions, thereby losing the ability to perform normal work and daily living activities^1, 2^. Thus, this trauma will lead to a heavy spirit and economic burden to both family and society. The elucidation of its mechanism and identification of an efficient strategy for the treatment of TBI represent an urgent need.

In addition to the uncontrollable and irreversible physical injury that leads to brain tissue damage, inflammation is a critical pathological process that aggravates the progression of TBI^1, 3, 4^. The triggers and participants of inflammation after TBI include native inflammatory cells (such as macrophages and microglial cells) in the central nervous system (CNS) as well as infiltrated peripheral blood immune cells^5, 6^. Neutrophils are the first peripheral immune cells to transmigrate across the blood-brain barrier (BBB) and infiltrate the brain after TBI, which is an important contributor to inflammatory injury of the brain during the acute phase^1, 7^. Several experimental results have indicated that the elimination of neutrophils was beneficial during the acute phase of brain injuries^8–10^. Therefore, one potential strategy is to halt brain inflammation by blocking neutrophil infiltration during the acute phase.

Metabotropic glutamate receptor 5 (mGluR5) is a group I metabotropic glutamate receptor, which exerts an important effect on inflammatory regulation after TBI. For example, Loane and Wang *et al*. reported that the the agonist of mGluR5, (RS)-2-chloro-5-hydroxyphenylglycine (CHPG), inhibits caspase-dependent apoptosis of neurons and suppresses inflammatory cytokine release from microglia^11, 12^. Movsesyan *et al*. found that the inactivation of mGluR5 also alleviates neuronal injury *in vitro* ^13^ and suppresses astrocytes that release proinflammatory cytokines or chemokines^14^. These studies provide important clues for understanding the mGluR5 function in regulating brain native cells that participate in inflammation and brain damage after TBI. However, it is not clear about the role of mGluR5 in the modulation of peripheral immune cells (such as neutrophils) during the course. Thus, in the present study, we focused on the effect of mGluR5 on peripheral neutrophil infiltration and associated brain damages in the acute phase of TBI, which is expected to provide insight regarding mGluR5 in TBI and novel clues for TBI therapy.

## Results

### MGluR5 deficiency protected mice against neurological dysfunction in the acute phase of TBI

**Figure 1:**
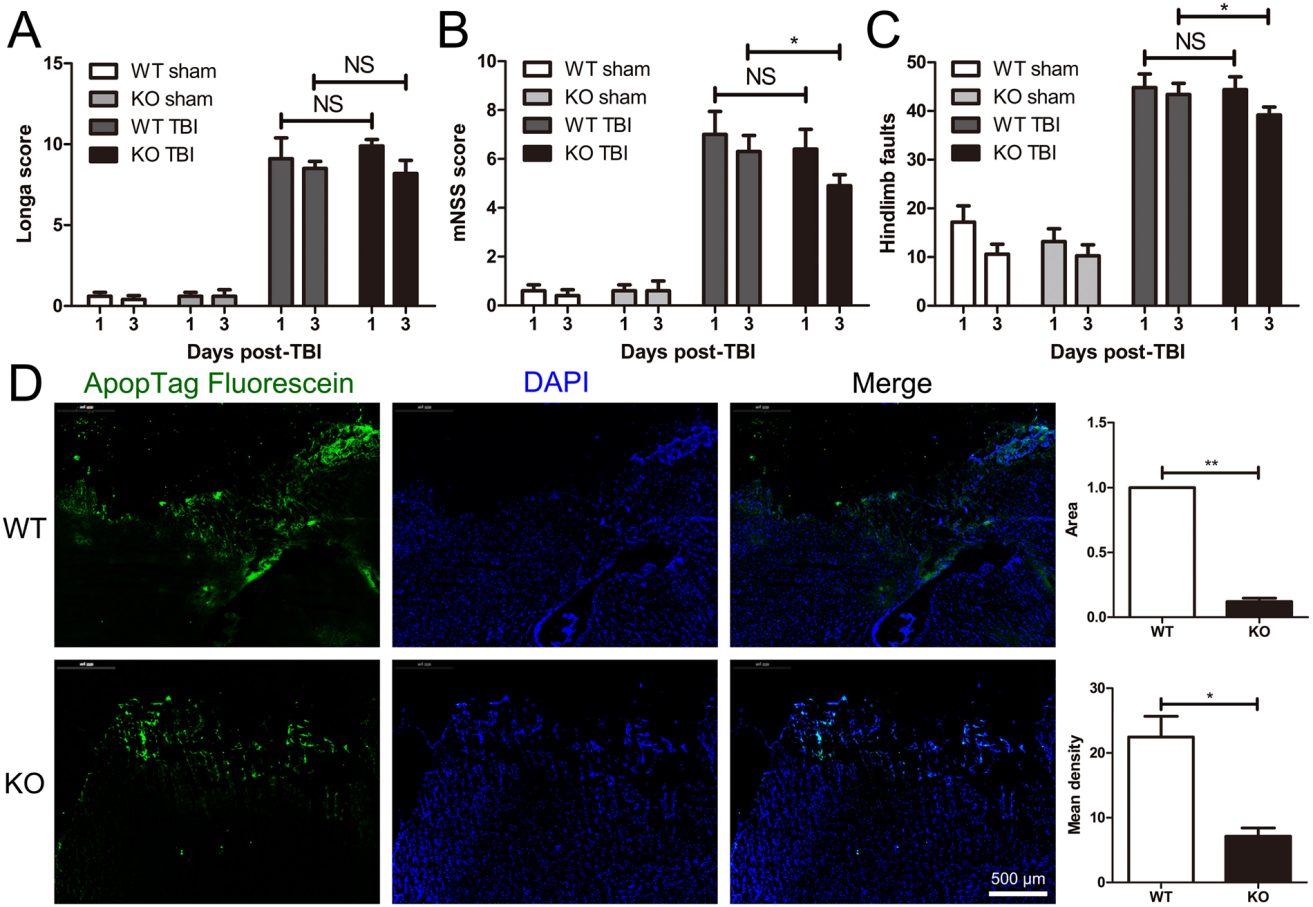
Knockout of mGluR5 attenuates neurological dysfunction in acute phase of TBI. Behavioral recovery of eight- to ten-week-old mGluR5 KO and WT mice were assessed using the Longa score test (**A**), modified neurological severity score (mNSS) test (**B**) and foot-fault test (**C**) on Days 1 and 3 post-TBI. (**D**) Apoptotic cells in brain sections at 24 h post-TBI were detected by ApopTag fluorescein assay. Nuclei were stained with DAPI. The positive fluorescence area and mean density analysis are presented. Data are presented as the mean ± standard error of the mean (NS indicates no significant difference, *P < 0.05, **P < 0.01, n = 5 per group).

In a mouse CCI-induced moderate TBI model, we evaluated the differences between eight-to-ten-week-old WT and mGluR5 KO mice with respect to behavioral recovery. The brain water content, breath rate and weight were not different between the WT and KO groups prior to the trauma (Supplementary Figure S1). The Longa score and foot-fault test were conducted to assess motor and balance function, whereas motor, sensory, reflex, and balance tests were covered in the modified neurological severity score. The Longa scale was initially used; however, there were no significant differences between the WT and KO groups on Day 1 or Days 3 after TBI (Fig. 1A). The modified neurological severity scores were nearly the same on Day 1 post-TBI; however, on Days 3, the mGluR5 KO mice exhibited a significantly lower modified neurological severity score than the WT (Fig. 1B). Differences in the foot-fault test were absent on Day 1; however, the hindlimb faults were fewer in the KO group than the WT on Days 3 after TBI (Fig. 1C). In addition, less apoptotic cells in brain sections of mGluR5 KO mice were observed at 24 h post-TBI when compared to that of WT mice (Fig. 1D). These results demonstrate that neurological dysfunction was less severe in the mGluR5 KO mice in the acute phase of TBI.

### MGluR5 deficiency suppressed neutrophil infiltration and inflammation in brain tissue after TBI

**Figure 2:**
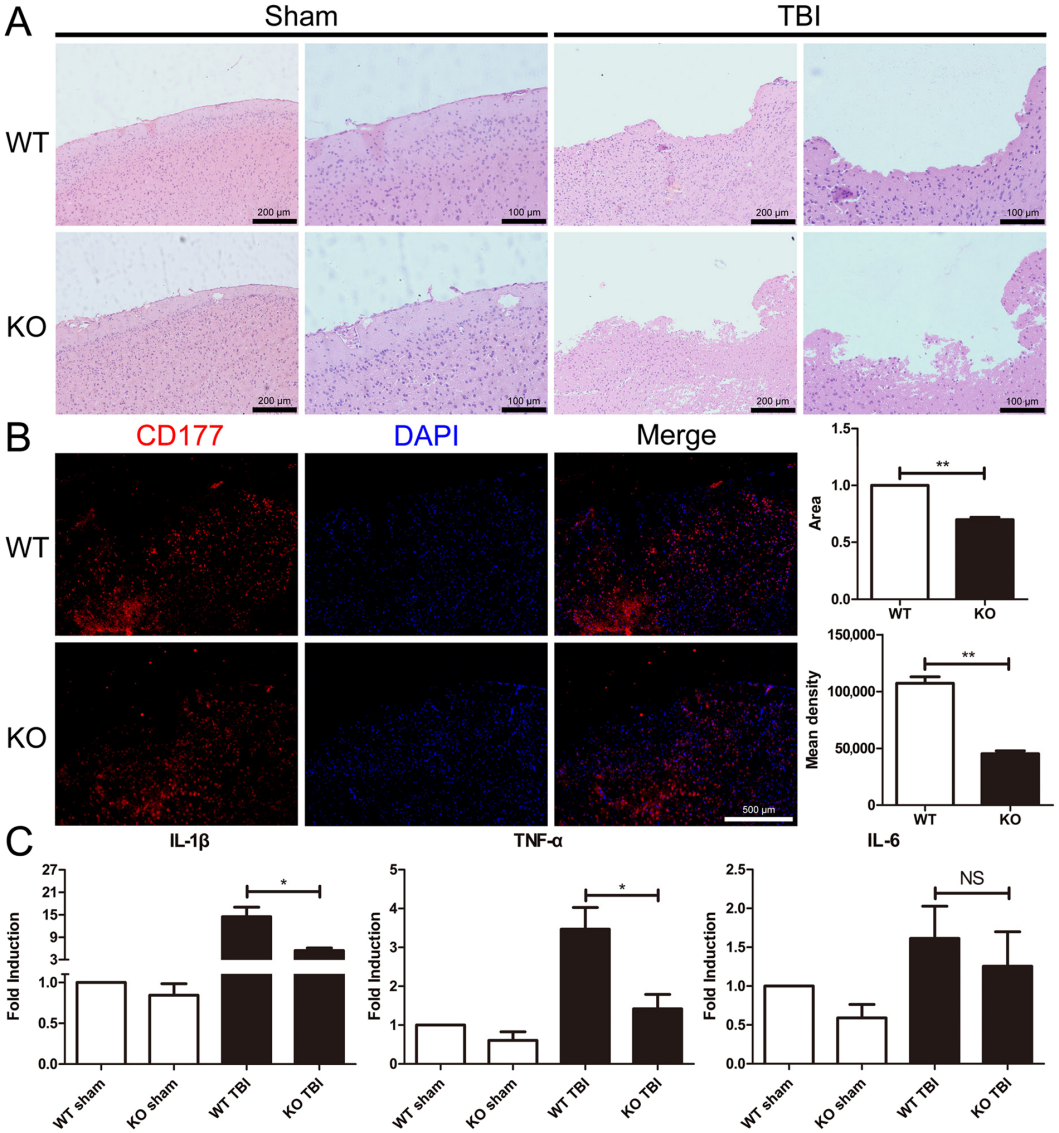
Knockout of mGluR5 suppresses neutrophil infiltration and inflammatory factor expression in brain tissues. (**A**) Brain sections of WT and KO mice were stained by HE at 24 h after sham operation and TBI. (**B**) Infiltrated neutrophils were tagged by CD177 in brain sections. Nuclei were stained with DAPI. The CD-177 positive fluorescence areas and mean density analysis were calculated by the software Image J. (**C**) IL-1β, TNF-α and IL-6 mRNA expression of the whole brain was detected via qRT-PCR. Data are presented as the mean ± standard error of the mean (*P < 0.05, **P < 0.01, NS indicates no significant difference; n = 5 per group).

To understand whether the effect of mGluR5 on regulating peripheral immune cells is involved in the protective effect of mGluR5 against TBI, we further observed the neutrophil infiltration and assayed inflammatory cytokine IL-1β and TNF-α expression in brain tissues. HE staining showed that the mGluR5 KO mice had substantially fewer infiltrated immunocytes near the site of injury in brain sections 24 h after TBI (Fig. 2A). Using CD177, a specific marker for neutrophils, we determined these infiltrated immune cells via immunofluorescence. The result showed that the CD177 positive cells were less in mGluR5 KO mouse slices than that in the WT mouse brain slices (Fig. 2B). This result is consistent with the results about inflammatory cytokine expressions. We found that the mRNA expression of IL-1β and TNF-α in brain tissues was significantly higher in the WT mice than the mGluR5 KO mice; although IL-6 was not significantly different (Fig. 2C). These findings suggest that mGluR5 KO could inhibit neutrophil infiltration and inflammation in the brain tissues after TBI.

### MGluR5 deficiency reduced the increase of BBB permeability induced by TBI

**Figure 3:**
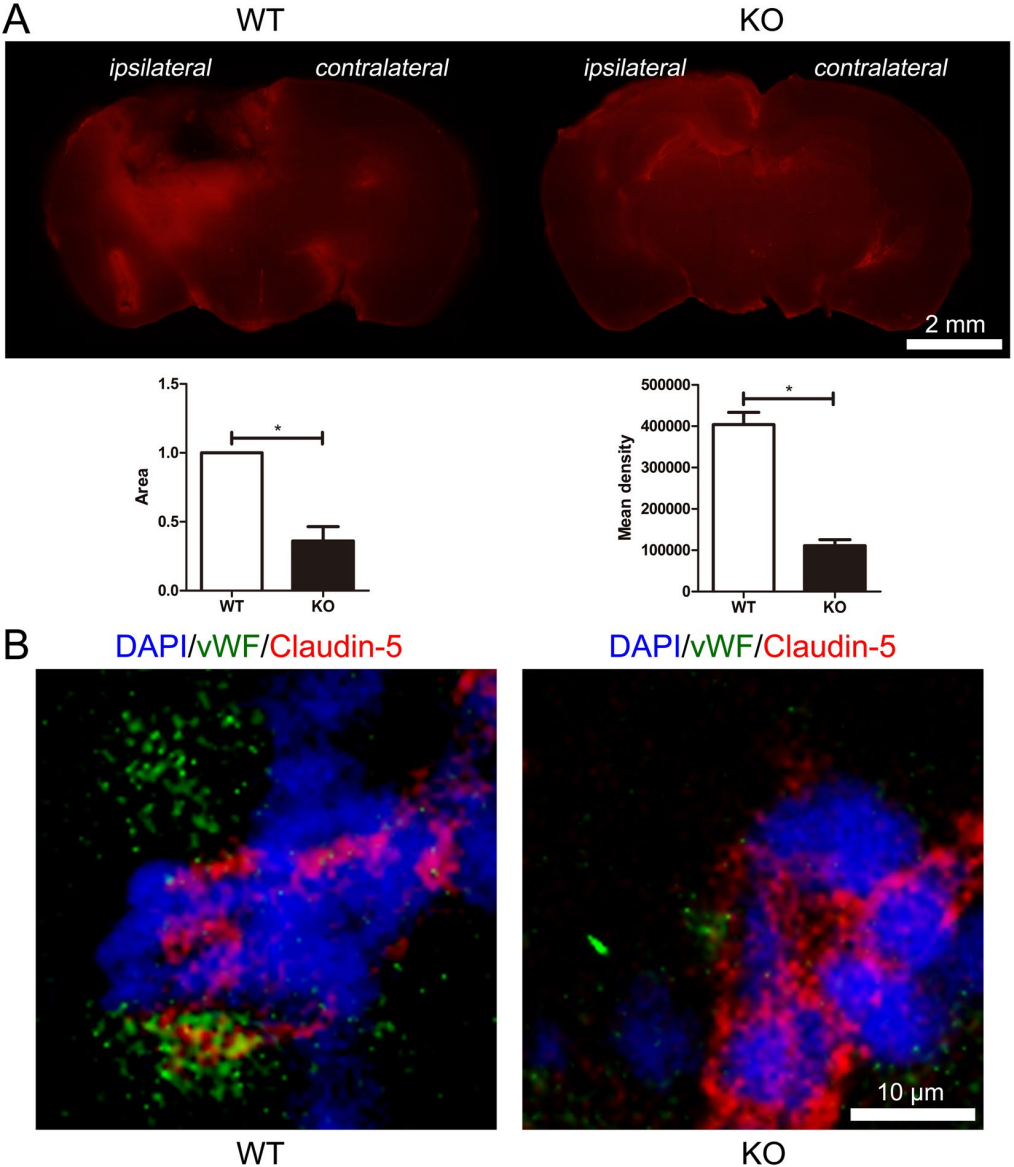
Knockout of mGluR5 reduces BBB permeability. (**A**) 24 h after TBI, the leakage of Evans blue was identified in brain sections at 2 h after injection. Area and mean density analysis are presented. Data are represented as the mean ± standard error of the mean (*P < 0.05). (**B**) Representative immunofluorescence images of tight junction protein claudin-5 localized at the periphery of vWF-positive endothelial cells. Nuclei were stained with DAPI.

Usually, BBB integrity is broken and permeability is increased post TBI, which provide an entrance for peripheral immune cells to infiltrate the brain parenchyma. Accordingly, we assessed whether mGluR5 deficiency affects BBB in the mouse TBI model. Plasma albumin passes the BBB after TBI, and Evans blue stain binds to serum albumin immediately after injection; thus, Evans blue stain accumulation is a reliable method to estimate the BBB permeability. As shown in Fig. 3A, at 24 h after TBI, an Evans blue assay indicated apparently less leakage of albumin in the brain slices of the mGluR5 KO mice compared with the WT. The expression of claudin-5, a tight junction protein that helps build the BBB, was subsequently examined using a fluorescence microscope (Fig. 3B). The mGluR5 KO mice had higher and more continuous claudin-5 expression than the WT in brain sections. These findings indicate that the normal integrity and permeability could be obviously maintained by mGluR5 deficiency, which may block neutrophil infiltration to a great extent.

### Expression of neutrophil-associated chemokine mRNAs in brain tissues were decreased by mGluR5 KO after TBI

**Figure 4:**
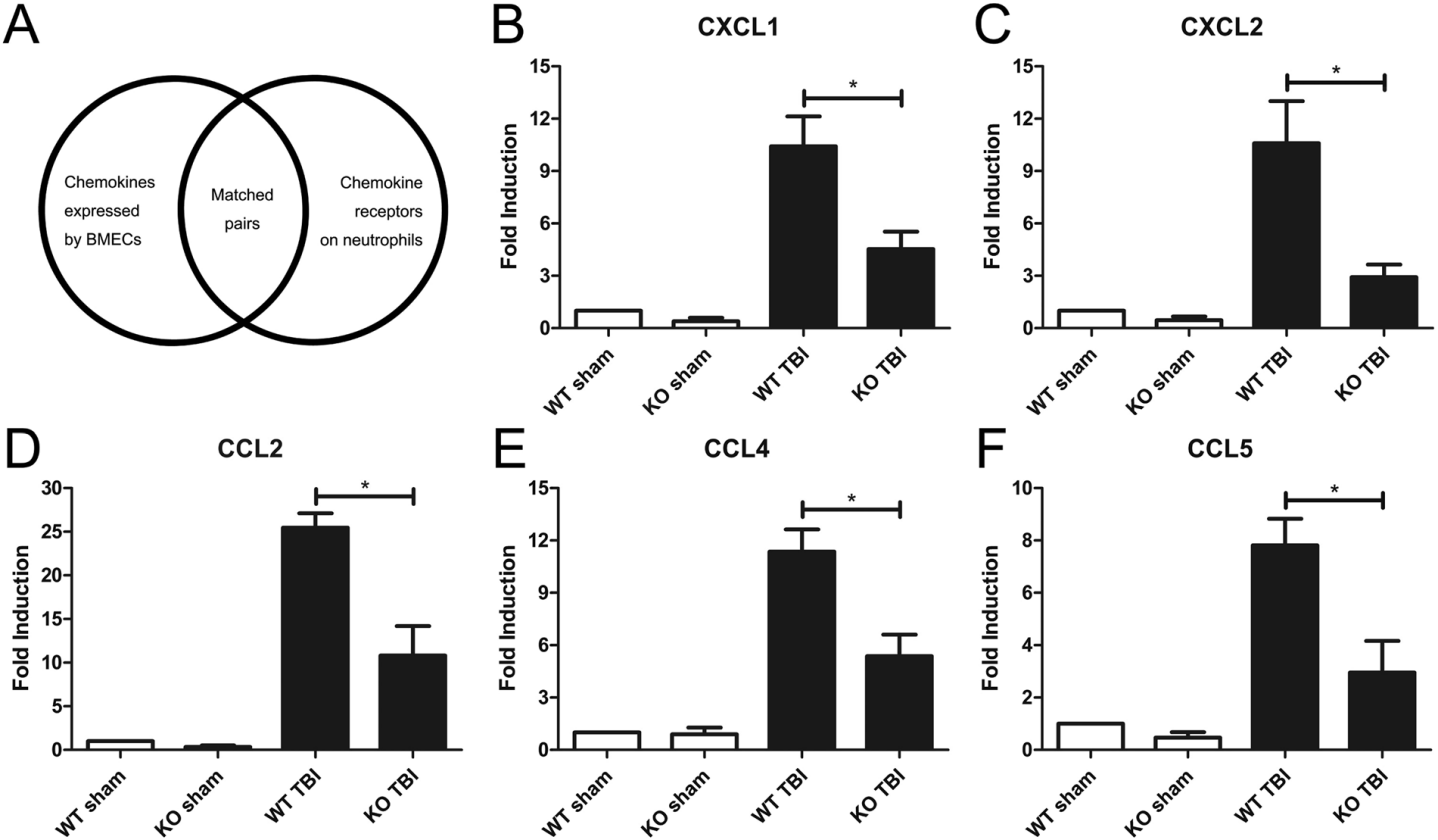
Knockout of mGluR5 downregulates the mRNA expression of chemokines in brain tissues. At 24 h post-TBI, main chemokines selected from matched pairs (**A**) involved in neutrophil trafficking were assayed via qRT-PCR. (**B**) mRNA expression of CXCL1. (**C**) mRNA expression of CXCL2. (**D**) mRNA expression of CCL2. (**E**) mRNA expression of CCL4. (**F**) mRNA expression of CCL5. Data are presented as the mean ± standard error of the mean (*P < 0.05).

Following the examination of the defensive construction against infiltration, we analyzed the factors related to the initiation and enhancement of neutrophil recruitment and infiltration after TBI. The transmigration of inflammatory cells from microvessels to the brain parenchyma is typically the result of an oriented movement of chemotaxis. Thus, we investigated the gene expression of the main brain microvascular endothelial chemokines that attract neutrophils in brain tissues (Fig. 4A). The receptor of CXCL1 and CXCL2 is CXCR2 on neutrophils, and CCL2, CCL4 and CCL5 pair with CCR1. At 24 h after CCI-induced TBI, both the WT and mGluR5 KO groups had higher expression of CXCL1 (Fig. 4B), CXCL2 (Fig. 4C), CCL2 (Fig. 4D), CCL4 (Fig. 4E), and CCL5 (Fig. 4F) in brain tissues than the sham groups. However, all five chemokines were significantly lower in the mGluR5 KO TBI group than in the WT TBI group (Fig. 4B–F). These findings suggest that the mRNA expression of neutrophil associated-chemokines in brain tissues were downregulated by the mGluR5 deficiency after TBI.

### Inactivation of mGluR5 inhibited neutrophil infiltration *in vitro*

**Figure 5:**
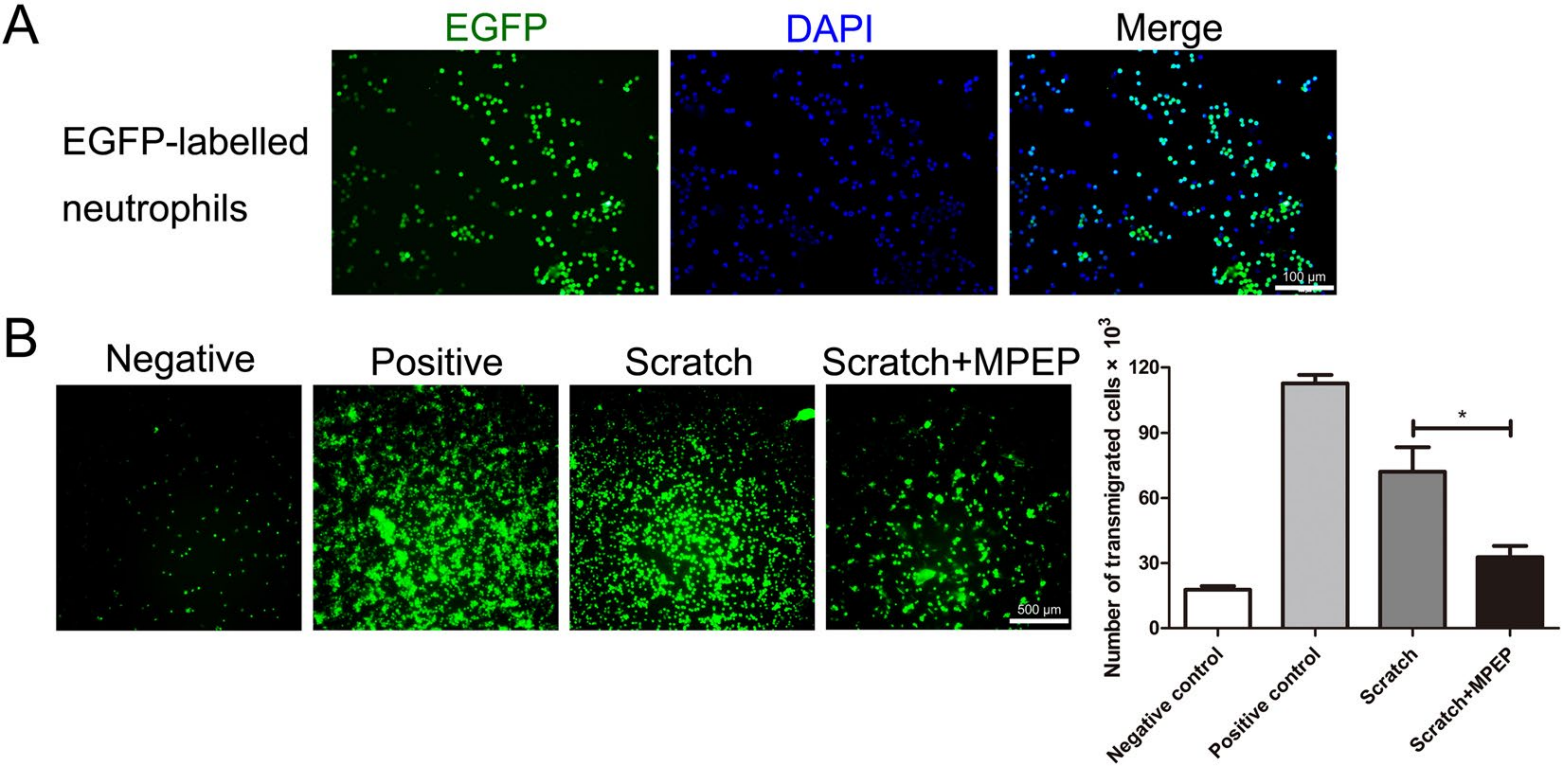
MGluR5 selective antagonist MPEP inhibits neutrophil transmigration through BBB *in vitro*. After mechanical scratch was performed on the monolayer of primary BMECs on transwells, EGFP-labeled neutrophils were added to the upper inserts of *in vitro* BBB models. In total, 50 μM MPEP was added 15 min prior to the mechanical scratch. The number of transmigrated cells were observed and calculated using a fluorescence microscope after 6 h. (**A**) Identification of EGFP-labeled neutrophils, which were obtained from the bone marrow of EGFP transgenic mice. Nuclei were stained with DAPI. (**B**) EGFP-labeled neutrophil transmigration through *in vitro* BBB models. “Negative” means the endothelial layer without scratch. “Positive” means scratches were performed almost everywhere on the confluent surface of the endothelial layer. The number of transmigrated cells is presented. Data are represented as the mean ± standard error of the mean (*P < 0.05).

Next, we constructed an *in vitro* model of BBB with brain microvascular endothelial cells (BMVCs) to confirm the suppressive effect of mGluR5 deficiency on neutrophil infiltration observed *in vivo*. Primary BMVCs were isolated to build the *in vitro* BBB models. When the monolayers reached confluency and the TEER were greater than 150 Ω·cm^2^ (Supplementary Figure S2), scratching it gently to mimic TBI mechanical injury, the *in vitro* BBB models were ready for use. EGFP-labeled neutrophils were subsequently extracted from the bone marrows of EGFP-transgenic mice (Fig. 5A). After pretreatment with the mGluR5 antagonist MPEP and mechanical scratches were performed on the monolayers of primary BMECs on transwells, EGFP-labeled neutrophils were added to the upper inserts of the *in vitro* BBB models. As shown in Fig. 5B, MPEP significantly reduced the number of transmigrated cells between the scratch group and the scratch pretreated with MPEP group. It indicates that mGluR5 inactivation suppresses neutrophil transmigration through the BBB *in vitro*.

### PKC pathway was involved in the regulation of mGluR5 on neutrophil-associated chemokine expressions in BMVCs

**Figure 6:**
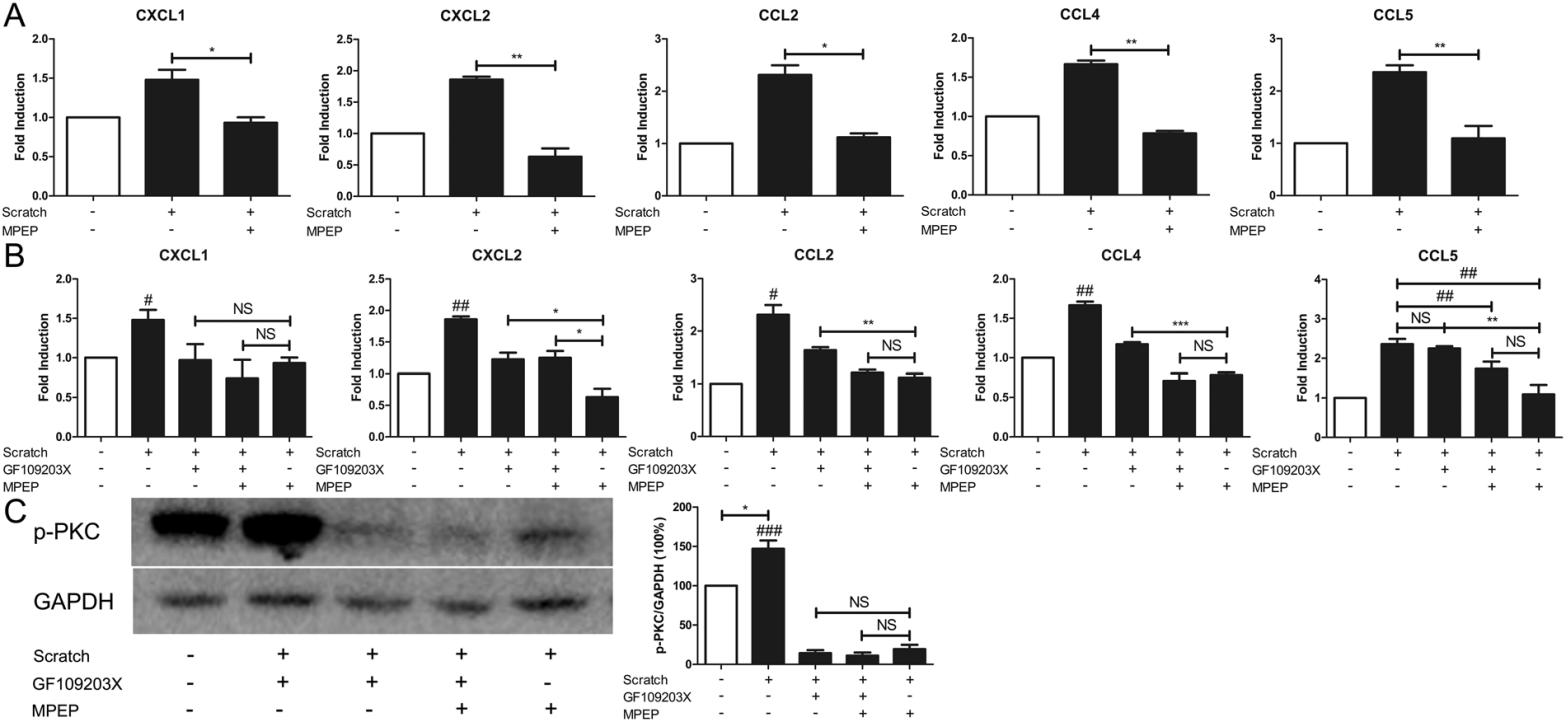
PKC signaling is involved in the regulation of mGluR5 on neutrophil trafficking. (**A**) *In vitro*, mechanical scratch (MS) was performed on mouse brain endothelial cells (bEnd.3, a BMEC cell line). In total, 50 μM MPEP was added 15 min prior to MS. Twenty-four h after MS, the mRNA expression of CXCL1, CXCL2, CCL2, CCL4 and CCL5 of bEnd.3 cells was examined via qRT-PCR. (**B**) PKC pathway, the main pathway downstream of mGluR5, was examined in a mechanical scratch (MS) model of bEnd.3 cells (a BMEC cell line) *in vitro*. In total, 1 μM PKC inhibitor GF109203X and 50 μM mGluR5 inhibitor MPEP were added 15 min prior to scratch. The mRNA expressions of CXCL1, CXCL2, CCL2, CCL4 and CCL5 of bEnd.3 cells were analyzed via qRT-PCR at 24 h post-scratch. (**C**) Western blot of p-PKC in bEnd.3 cells at 24 h post-MS and its quantitative analysis. Data are presented as the mean ± standard error of the mean (*P < 0.05, **P < 0.01, ***P < 0.001, ^#^P < 0.05 relative to the other scratch groups, ^##^P < 0.01 relative to the other scratch groups, ^###^P < 0.001 relative to the other scratch groups, NS indicates no significant difference).

Since BMVCs are main component of BBB and will secrets chemokines to initiate neutrophil infiltration, we further confirmed the mGluR5 deficiency-mediated suppression of neutrophil-associated chemokine expressions *in vitro* and investigated its molecular mechanism. Using bEnd.3, a murine brain endothelial cell line, we developed a mechanical scratch cell model that mimics TBI-induced traumatic BMEC injury *in vitro*. Cultures were pre-treated with the mGluR5 selective antagonist MPEP to inactivate mGluR5 post injury. We subsequently examined the same mRNA expression of the chemokines in the mouse TBI models previously described. As shown in Fig. 6A, MPEP significantly suppressed the scratch-induced expression levels of CXCL1, CXCL2, CCL2, CCL4 and CCL5 24 h post-injury compared with the untreated injured cells. The PKC pathway is the main downstream of mGluR5. Then, we examined whether PKC pathway inhibitor GF109203X will mimic the effect of mGluR5 inactivation on these chemokine expressions. Figure 6B showed that these mRNA expressions of chemokines including CXCL1, CXCL2, CCL2 and CCL4 were all significantly decreased in GF109203X pretreated groups than the untreated injured group. These mRNA expressions of CXCL1, CCL2, CCL4 and CCL5 had no significant difference between GF109203X plus MPEP pretreated groups and MPEP pretreated groups. In Fig. 6C, the mechanical scratch increased the protein expression of p-PKC, which could be inhibited by GF109203X and/or MPEP. These results suggest that PKC pathway is involved in the regulation of mGluR5 on neutrophil-associated chemokine expressions. However, there was no significant difference in CCL5 expressions between the GF109203X pretreated and untreated injured groups. This indicates that another pathway may mediate the downregulation of CCL5 by MPEP, which needs further investigation.

### MGluR5 deficiency downregulated most chemokine and chemokine receptor expression in neutrophils rather than affecting the number of neutrophils in blood or bone marrow

**Figure 7:**
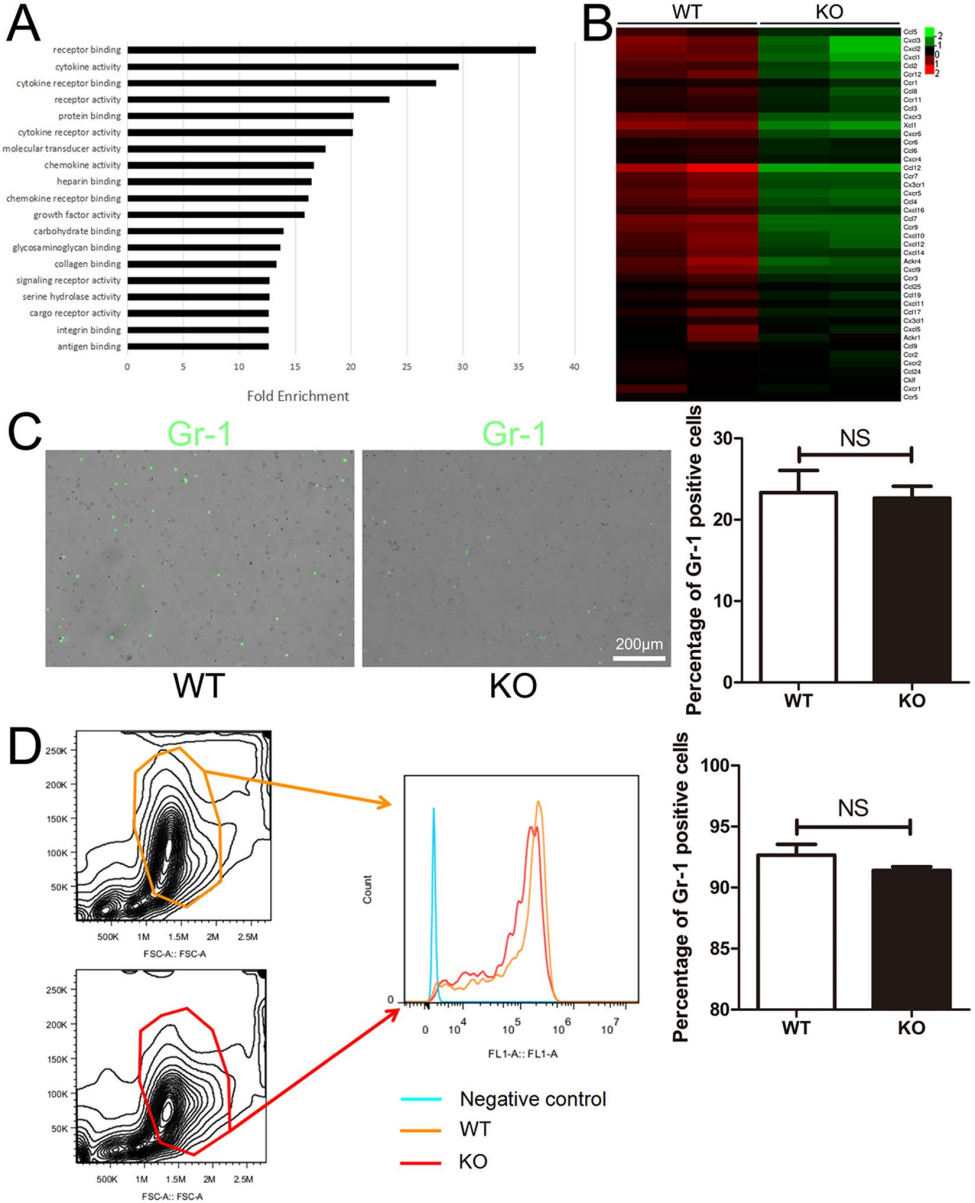
Knockout of mGluR5 downregulates most chemokines and chemokine receptors of neutrophils rather than the number of neutrophils in blood or bone marrow. (**A**) Apparent changed pathways between WT and mGluR5 KO neutrophil samples and the fold enrichments of WT groups compared with mGluR5 KO groups. (**B**) Hierarchical cluster analysis of array data. Each column represents a sample, and each row represents a single mRNA. The color scale shown on the right illustrates the relative expression level of an mRNA in a specific slide: green indicates negative values (down-regulation), which represent low relative expression levels; red indicates positive values (up-regulation), which represent high relative expression levels; black indicates zero (no change). Gr-1 positive cells from peripheral blood (**C**) were observed under a fluorescence microscope, whereas cells from bone marrow (**D**) were detected via flow cytometry. The percentage of Gr-1 positive cells is presented. Data represent the mean ± standard error of the mean (NS indicates no significant difference).

To decipher whether neutrophils themselves were altered before infiltration under a traumatic brain injury condition, microarray was performed on bone marrow-derived neutrophils from WT and mGluR5 KO mice separately. The pathway analysis indicated that several pathways changed when mGluR5 was knocked out, particularly cytokine and chemokine related activities (Fig. 7A). As shown in Fig. 7B, some important chemokine and chemokine receptor related genes significantly decreased in the neutrophils isolated from mGluR5 KO groups (Fig. 7B). To investigate whether the number of neutrophils changes prior to infiltration, we isolated the peripheral blood and counted the Gr-1 (murine neutrophil specific marker) positive cells via immunofluorescence. We subsequently calculated the Gr-1 positive neutrophils in bone marrow from the femur and fibula of mice via flow cytometry. There were no significant differences between the WT and KO mice as indicated in Fig. 7C–D. Taken together, it confirms that the mGluR5 deficiency plays an important role in suppressing the chemotaxis of neutrophils rather than reducing the number of neutrophils in mouse blood or bone marrow.

## Discussion

In this study, we focused on the role of mGluR5 in regulating peripheral immune cells in a mouse TBI model and demonstrated that the inhibition of neutrophil infiltration is involved in the mGluR5 deficiency-mediated protection against inflammation and brain damage post TBI. Here, for the first time, we address the important regulatory effect of mGluR5 on peripheral immune cells to affect the progression of brain inflammation and the outcome of TBI as well as elucidate its associated mechanisms.

As one of glutamate endogenous receptor, mGluR5 is found to be involved in several neurological disorders including schizophrenia, Parkinson’s disease (PD) and stroke^15, 16^. Recently, more and more evidence indicates that mGluR5 plays an important role in TBI. For example, activation of mGluR5 reduces the neuronal apoptosis under the condition of oxygen-glucose deprivation^17^ and inhibits microglial associated inflammation^18^. Furthermore, Loane and Wang *et al*. reported that activation of mGluR5, (RS)-2-chloro-5-hydroxyphenylglycine (CHPG) injected intracerebroventricularly, inhibits caspase-dependent apoptosis of neurons and suppresses inflammatory cytokine release from microglias^11, 12^. However, inactivation of mGluR5 also alleviates neuronal injury *in vitro* ^13^ and suppresses astrocytes releasing proinflammatory cytokines or chemokines^14^. Although these results are somewhat controversial, they provide a vital clue for mGluR5 function in TBI. It has been reported that mGluR5 is expressed both on cell surface and intracellular endoplasmic reticulum and nuclear membranes, which can activate distinct signaling cascade^19^. Most of these previous studies used mGluR5 agonists or antagonists in animal models, some of which are nontransported and impermeable so that they can only regulate cell surface mGluR5. In this study, we knocked out mGluR5 in a mouse TBI model and found that the mGluR5 deficiency significantly protected mice against neurological dysfunction in the acute phase of TBI. When compared with the nontransported and impermeable mGluR5 agonists or antagonists, global knockout of mGluR5 inactivates both cell surface and intracellular mGluR5 rather than only the surface mGluR5. Thus, to some extent, our findings provide some novel information to understand the complete role that mGluR5 plays during TBI.

In TBI, after initial mechanical trauma causes direct damage, microglia and astrocytes begin a cascade of immune events heralded by the release of damage-associated molecules^14, 20^. These molecules activated resident glia, also attracted peripheral WBCs to initiate and promote inflammation and perpetuate neural injury. Neutrophils are the most prevalent WBCs in blood and serve as the first line of defense against injuries, which is found to enter brain in minutes post TBI. Here, we demonstrated that being companied with improvement of neurological function, mGluR5 deficiency dramatically decreased brain neutrophil infilatraion and inflammatory cytokine expressions after TBI. It confirms the important effect of mGluR5 on inflammatory regulation in TBI and suggests that this regulation is not limited to brain resident cells, it is also to peripheral immune cells.

In normal, the BBB protects the brain and maintains the homeostasis. In TBI, injury to the cerebral vasculature breaks the BBB and allows entries of immune cells and stimulates inflammation. Here, when we focused on the inhibitory effect of mGluR5 deficiency on neutrophil infiltration, we noticed that mGluR5 knockout significantly alleviated the damage of BBB integrity and permeability induced by TBI. Using an *in vitro* BBB model, we confirmed that this modulation of BBB is involved in the mGluR5 deficiency-mediated suppression of neutrophil infiltration. These data are consistent with the evidence that mGluR5 on brain endothelial cells may modulate the permeability through phosphorylated vasodilator-stimulated phosphoprotein in a hypoxic mouse model^21^. Besides the leaky BBB provides the entrance, upon the initiation of infiltraion, neutrophils are attracted to the endothelium by chemokines. Here, we presented that the neutrophil-associated chemokine expressions in BMECs and brain tissues were significantly decreased when mGluR5 was knocked out or inactivated. Further investigation indicated that mGluR5 deficiency blocking the PKC signaling resulted in the inhibition of these chemokine expressions. MGluR5 couples to G_q_/G_11_ and activates phospholipase Cβ, which results in the hydrolysis of phosphoinositides and generation of inositol 1,4,5-trisphosphate and diacylglycerol. This classical pathway leads to calcium mobilization and PKC activation^22^. Besides PKC signaling, mGluR5 also activates some PKC-independent pathways, including ERK and Akt^23^. Although further investigation needs to detect whether these signal pathways are also involved in the process, these results demonstrate a novel function of mGluR5 in TBI.

To exclude the possibility that the less infiltrated neutrophils in brain was resulted from reduced neutrophil number in circulation, we detected the effect of mGluR5 deficiency on neutrophils themself. The results showed that mGluR5 deficiency down-regulated chemokine receptor expressions of neutrophils but did not affect the number of neutrophils in blood or bone marrow. This finding confirmed that mGluR5 deficiency suppressed neutrophil infiltration rather than neutrophil generation. It is known that mGluR5s are relatively highly expressed in the CNS compared with peripheral tissues; however, some researchers have demonstrated that mGluR5 was developmentally regulated^24^. The expression of mGluR5 in peripheral immune cells, including neutrophils, T cells, and dendritic cells, may be induced^25, 26^ and significantly regulate the activities of these cells in cases of pathological condition^27–29^. Being consistent with these reports, we found that mGluR5 expression in neutrophils could be induced under a TBI condition within 24 hours (Supplementary Figure S3). Although it is not clear how the increased mGluR5 regulates neutrophil, this result supports the impact of mGluR5 deficiency on neutrophil chemokine expressions and neutrophil infiltration.

Taken together, the present study demonstrates mGluR5 deficiency inhibits neutrophil infiltration after TBI via maintaining the BBB permeability and reducing the chemotaxis of neutrophils. This shows a novel aspect of mGluR5 function in inflammation and brain damage in TBI, which may provide potential novel strategies for TBI therapy.

## Materials and Methods

### Animals

Global mGluR5 homozygous KO mice and their WT littermates were purchased from the Model Animal Research Center of Nanjing University^30^. In brief, congenic global mGluR5 KO mice on a C57BL/6 background were generated by backcrossing global mGluR5 KO on a mixed (129-Steel × C57BL/6) genetic background to C57BL/6 mice for 13–15 generations. Heterozygous crossbreeding was used to generate global mGluR5 KO mice and their WT littermates. EGFP transgenic mice were obtained from the Department of Laboratory Animal Science, College of Basic Medical Sciences, Third Military Medical University^31^. All mice were maintained in a pathogen-free animal facility under 12 h light/dark conditions with food and water available ad libitum at the Animal Center, Research Institute of Surgery and Daping Hospital, Third Military Medical University. All experimental procedures were approved by the Animal Care Committee of the Third Military Medical University and all experiments were performed in accordance with relevant guidelines and regulations.

### TBI model

An automatic brain impact machine (LinTech, Monrovia, CA, USA) was used to induce moderate TBI in both WT and mGluR5 KO mice as previously described^32, 33^ with modifications. Briefly, after the mice were anesthetized with an intraperitoneal injection of 1.5% sodium pentobarbital and fixed in a stereotaxic frame, the scalp was incised, and a dental drill was used to perform a craniotomy with a trephine bit (ø = 3 mm) on the left motor cortex (anterior-posterior 2 mm, medial-lateral 2 mm from bregma). The cortex surface at the center of the craniotomy was targeted vertically using a metal tip (ø = 2.5 mm). When the coarse adjustment and fine adjustment were completed, the machine induced a TBI by retracting the tip upward by 2 cm, followed by a down stroke (velocity: 1.5 m/s, deformation depth: 2 mm, duration: 120 ms). The wound was immediately sutured, and the mice were returned to the animal facility.

### Neurological deficit scoring

The Longa scale^34^, a modified neurological severity score (mNSS)^35^ and a foot-fault test^11^ were used to assess the neurological deficits of each animal prior to sacrifice by two observers blinded to the treatment. Motor, sensory, reflex, and balance tests were included. A higher score or more foot faults represented a more severe injury. The number of foot faults for the right hindlimb was recorded over 50 steps while the mice crossed a narrow wooden beam (6 mm wide and 120 mm in length). All mice were trained to perform the tests for 3 days prior to TBI.

### HE staining

The mice were anesthetized 24 h after injury and transcardially perfused with saline, followed by 4% paraformaldehyde. The brains were immediately removed and post-fixed in 4% paraformaldehyde for 24 h. Paraffin-embedded sections (10 μm thick) were stained with hematoxylin and eosin (HE) and observed under a microscope (Olympus IX-81; Olympus, Tokyo, Japan).

### Immunofluorescence

Immunofluorescence analysis were conducted as previously described^36, 37^. After quenching the endogenous peroxidase activity and blocking the nonspecific binding, the sections were detected the ApopTag plus *in situ* apoptosis fluorescein detection assay kit (Millipore Co., Billerica, MA,USA) or allowed to react with the primary antibodies (a rabbit anti-CD177 antibody conjuga, a neutrophil marker, 1:200; ZSGB-BIO, Beijing, China; rabbit anti-vWF, an endothelial cell marker; 1:200; Bioss, Beijing, China; or goat anti-claudin-5 (a tight junction protein; 1:200; Santa Cruz Biotechnology, Dallas, TX, USA)) at 4 °C overnight. Those sections with primary antibody incubation were subsequently washed, incubated with Alexa Fluor 488- or Alexa Fluor 555-conjugated secondary antibodies (1:200; Thermo Fisher Scientific, Rockford, IL, USA) for 1 h, observed under a fluorescence microscope (Olympus IX-81; Olympus, Tokyo, Japan), and photographed with nuclei stained with DAPI. Murine peripheral blood was stained with the specific marker Gr-1 conjugated Alexa 488 (1:200; eBioscience, San Diego, CA, USA) diluted with PBS that contained 2% BSA for 45 min at 4 °C; they were observed and calculated under a fluorescence microscope (Olympus IX-81; Olympus, Tokyo, Japan). The software Image J (NIH, Bethesda, MD, USA) was used to analyze the average fluorescence area and mean density to indicate the positive cells.

### Evans blue assay

An Evans blue assay was conducted as previously described^38^. The mice were anesthetized, and 2% Evans blue dye (4 ml/kg; Sigma Aldrich, St. Louis, MO, USA) was intravenously injected and allowed to circulate for 2 h. The mice were then transcardially perfused with physiological saline until no blue color was evident in the effluent. The brain was removed and sliced into 2 mm thick sections. Extravasated Evans blue was visualized using a fluorescence microscope (Olympus DP-73; Olympus, Tokyo, Japan) in fixed, frozen cryostat sections. The area and mean density of fluorescence micrographs of extravasated Evans blue dye were calculated using the software ImageJ (NIH, Bethesda, MD, USA).

### Cell cultures

Neutrophils were isolated from the femur and fibula of mice as previously described^39^ with modifications. Murine bone marrow was dispersed in HBSS (without Ca^2+^ and Mg^2+^) with 0.5% fetal calf serum (FCS) (HBSS/FCS), strained through a 100-μm cell strainer (Falcon; Corning, NY, USA), and centrifuged (1000 g, 30 min, room temperature) over 2-layer (0/62.5%) Percoll gradient in HBSS. Mature EGFP-labelled neutrophils were obtained from the lower part of 62.5% Percoll (purity, 80–90% by cytospin), and contaminating red blood cells were removed via ammonium chloride lysis. Finally, the cells were washed twice in HBSS/FCS. The attained neutrophils were diluted in RPMI-1640 media with 10% (v/v) fetal bovine serum (FBS) to a final concentration of 10^6^/ml and cultured at 37 °C in an atmosphere of 5% (v/v) CO_2_ for the indicated time.

Brain microvascular endothelial cells (BMVCs) were isolated from mouse brains as described^40^. In brief, the brains were removed from the mice and stored in Dulbecco’s modified Eagle’s medium (DMEM) on ice. Meninges were carefully removed from the forebrains, and the forebrains were mashed with forceps and minced into small pieces of approximately 1 mm^3^, followed by digestion with 2 mg/ml collagenase/dispase (Roche Life Science, Indianapolis, IN, USA) in DMEM for 45 min at 37 °C (occasionally shaking). Cold DMEM was added to the homogenate and centrifuged at 1000 g, 4 °C for 8 min. The pellet was resuspended in 25% bovine serum albumin (BSA) and centrifuged at 1000 g, 4 °C for 20 min. The resulting suspension that contained the vascular component was filtered through a 59 μm nylon mesh. BMEC cultures were washed and maintained in Endothelial Cell Medium (ECM; ScienCell Research Laboratories, San Diego, CA, USA) supplemented with 100 mg/ml heparin and 4 mg/ml puromycin^41^ and maintained in humidified air that contained 5% (v/v) CO_2_ at 37 °C in an incubator. After 24 h, the culture medium was replaced, and puromycin was removed from the ECM. The isolated cells were identified with vWF, an endothelial cell marker, via immunofluorescence to ensure the purity of the cells. When the cultures reached 80% confluency, the purified BMECs were used to construct *in vitro* BBB models.

Murine brain endothelial cells (bEnd.3), which have been recognized to present brain endothelium-like properties, were obtained from American Type Culture Collection (ATCC). This cell model is an appropriate choice to investigate blood-brain barrier function^42^. The cells were cultured in DMEM supplemented with 10% (v/v) FBS and antibiotics (100 U/ml penicillin and 100 μg/ml streptomycin) in humidified air that contained 5% (v/v) CO_2_ at 37 °C in an incubator.

### qRT-PCR

Total RNA from tissues or cultured cells was isolated using TRIzol Reagent (Invitrogen, CA, USA). Two μg of template RNA was used to synthesize cDNA using a reverse transcription kit (TaKaRa, Dalian, China). The following primers were used for qPCR: IL-1β: forward, 5′-ACT GTT TCT AAT GCC TTC CC-3′′; reverse, 5′-ATG GTT TCT TGT GAC CCT GA-3′; TNF-α: forward, 5′-CTG TGA AGG GAA TGG GTG TT-3′; reverse, 5′-TCA CTG TCC CAG CAT CTT GT-3′; IL-6: forward, 5′-AGT TGC CTT CTT GGG ACT GA-3′; reverse, 5′-TCC ACG ATT TCC CAG AGA AC-3′; CXCL1: forward, 5′-ATC CAG AGC TTG AAG GTG TTG-3′; reverse, 5′-GTC TGT CTT CTT TCT CCG TTA CTT-3′; CXCL2: forward, 5′-ATG CCT GAA GAC CCT GCC AAG-3′; reverse, 5′-GGT CAG TTA GCC TTG CCT TTG-3′; CCL2: forward, 5′-GCT GAC CCC AAG AAG GAA TG-3′; reverse, 5′-GTG CTT GAG GTG GTT GTG GA-3′; CCL4: forward, 5′-CCA GGG TTC TCA GCA CCA A-3′; reverse, 5′-GCT CAC TGG GGT TAG CAC AGA-3′; CCL5: forward, 5′-CTC ACC ATA TGG CTC GGA CA-3′; reverse, 5′-CTT CTC TGG GTT GGC ACA CA-3′; GAPDH: forward, 5′-CAT CAC TGC CAC CCA GAA GA-3′; reverse, 5′-CAG ATC CAC GAC GGA CAC AT-3′; and mGluR5: forward, 5′-GTC CTG GCC CAC TGA CGA-3′; reverse, 5**′**-GGT CAC CCC ATC GAA GAT AC-3′. Quantitative PCR was performed using an ABI 7500 thermocycler that employed an SYBR Premix Ex Taq^TM^ II Kit (TaKaRa, Dalian, China). The mRNA expression levels of interest were normalized to the level of the endogenous control using the 2^−ΔΔCt^ method.

### Western blot

The whole-cell protein lysates were achieved and western blot analysis were performed as described previously^43^. The primary antibodies p-PKC or mGluR5 (Abcam, Cambridge, MA, USA) were used. At final, the Blots were scanned and analyzed by Image J software. The normalized band intensities against corresponding GAPDH were calculated for precise comparison.

### Mechanical scratch model on BMECs

Mechanical scratches were performed to mimic traumatic injury on brain microvascular endothelial cells according to a previously described method^44, 45^. In brief, a traumatic injury was induced on cultured brain endothelial cells (bEnd.3) using a 10 μl pipette tip. We made 4 crosswise lines in the cell culture holes symmetrically. Moreover, the injury lines were produced on the confluent surface of the endothelial layer with equal distances between the scratches, and detached cells were removed by washing with DMEM.

### Blood-brain barrier model and transmigration assay

Blood-brain barrier models were performed as previously described^46^ with modifications. Briefly, BMECs (5 × 10^5^ cells/cm^2^) were seeded on the upper side of a collagen-coated polyester membrane of transwell inserts (polyester membranes, 3 μm pore size, ø = 6.5 mm; Corning, NY, USA) placed in the holes of 24-well culture plates. The BBB models were maintained in ECM with media replacement that occurred every other day until the monolayers reached confluency.

Transmigration assays were conducted as previously described^47^ with modifications. After mechanical scratches (2 symmetrical crosswise lines) were performed on the monolayers of BMECs, EGFP-labeled neutrophils (1 × 10^6^ cells per well) were added to the upper inserts of the transwell system of *in vitro* BBB models. Over a 6-h period at 37 °C, the number of transmigrated cells from the lower chambers were observed using a fluorescence microscope (Olympus DP-73; Olympus, Tokyo, Japan) and calculated by the haemacytometer. In the experiment, the endothelial layer without scratch served as the negative control while the endothelial layer with scratch everywhere served as the positive control.

### Microarray assay

Total RNA of the neutrophils isolated from the bone marrows of the WT or mGluR5 KO mice was prepared using TRIzol Reagent (Invitrogen, CA, USA). Standard cDNA synthesis, probe labeling, hybridization, and scanning were performed using the standard protocols in CapitalBio Technology (Beijing, China). Expression profilings were conducted using Affymetrix Mouse Genome 430 2.0 arrays (Affymetrix, CA, USA). Four samples were assayed, including 2 samples from the WT mice and 2 samples from the mGluR5 KO mice. Microarray visualization of the GO pathway analysis and data filtering of chemokine related genes were accomplished by the specific manager of CapitalBio Technology.

### Flow cytometry

The isolated bone marrow neutrophils were stained with the specific marker Gr-1 conjugated Alexa 488 (1:200; eBioscience, San Diego, CA, USA) diluted with PBS that contained 2% BSA for 45 min at 4 °C. Flow cytometry data of the bone marrow neutrophils were acquired with a FACSCanto II (BD Biosciences, San Jose, CA, USA) and analyzed with FlowJo software (Tree Star, Ashland, OR, USA).

### Statistical analysis

The obtained data were presented as the mean ± SEM for all independent experiments performed in triplicate. One-way or two-way analysis of variance (ANOVA) followed by a post hoc Bonferroni evaluation were used for multiple groups to determine significant differences. Student’s t test was used to test the differences between two groups. Differences were considered statistically significant when the p-value was < 0.05.

## Electronic supplementary material

Supplementary information
