# Supplementary material for: Metabotropic glutamate receptor 5 deficiency inhibits neutrophil infiltration after traumatic brain injury in mice
Source: Sci Rep. 2017 Aug 30;7:9998. doi: 10.1038/s41598-017-10201-8 (PMC5577182; doi:10.1038/s41598-017-10201-8)
Supplement: Supplementary file 1 — Supplementary information [file 41598_2017_10201_MOESM1_ESM.doc]

**Metabotropic glutamate receptor 5 deficiency inhibits neutrophil infiltration after traumatic brain injury in mice**

Ting Yang^1^, Yang-Wuyue Liu^1^, Li Zhao^1^, Hao Wang^2^, Nan Yang^3^,

Shuang-Shuang Dai^1,3^ & Fengtian He^1^

*^1^Department of Biochemistry and Molecular Biology , Third Military Medical University, Chongqing 400038, PRC. ^2^Department of Neurosurgery, Research Institute of Surgery and Daping Hospital, Third Military Medical University, Chongqing 400042, PRC. ^3^Molecular Biology Center, State Key Laboratory of Trauma, Burn, and Combined Injury, Daping Hospital, Third Military Medical University, Chongqing 400042, PRC. T.Y. and Y.-W.L contributed equally to this study. Correspondence and requests for materials should be addressed to S.-S.D. and F.H. (email: daiyuyang268@aliyun.com and hefengtian06@aliyun.com)*


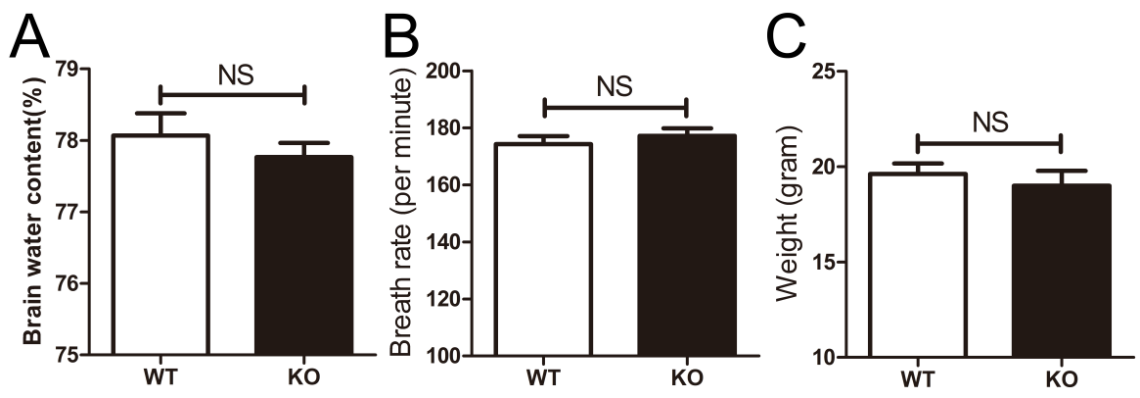


**Supplementary Figure S1:** **Brain water content (A), breath rate (B) and weight (C) of the WT and KO prior to TBI.** Data are presented as the mean±standard error of the mean (NS indicates no significant difference; n=5 per group).

**Supplementary Figure S2: TEER of in vitro BBB models.**


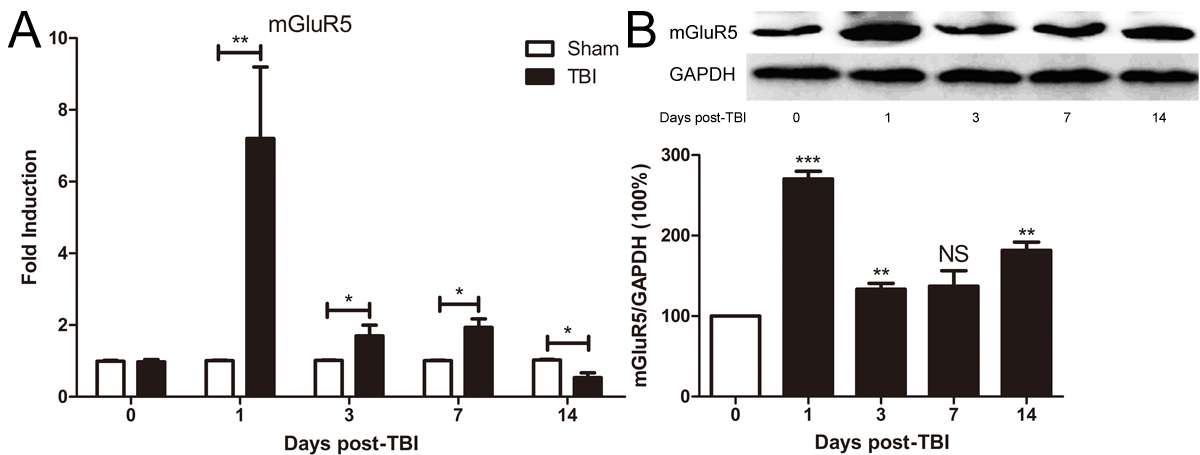


**Supplementary Figure S3: The mRNA (A) and protein (B) expression of mGluR5 of neutrophils from bone marrow prior to trauma and on Days 1, 3, 7, and 14 post-TBI.** Data are presented as the mean±standard error of the mean (*P<0.05 vs. Sham group, **P<0.01 vs. Sham group, ***P<0.001 vs. Sham group, NS indicates no significant difference vs. Sham group; n=5 per group).

**A**

**
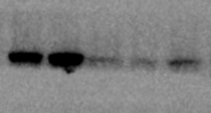
**

**p-PKC**

**(80KDa)**


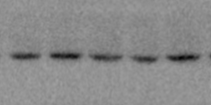
**B**

**GAPDH**

**Supplementary Figure S4: Full gels of Figure 6C.** The full gels of p-PKC (A) and GAPDH (B) were presented. The dilution ratio of the antibody of p-PKC is 1:500.

**A**

**
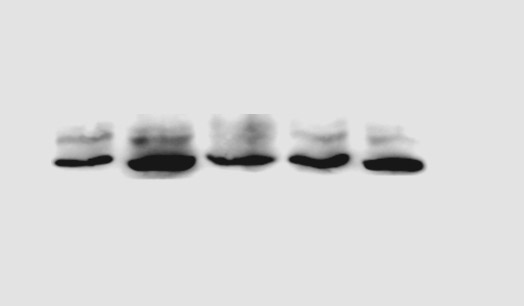
**

**mGluR5**

**(132KDa)**

**B**


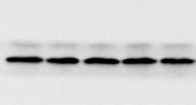


**GAPDH**

**Supplementary Figure S5: Full gels of Supplementary Figure S3 B.** The full gels of mGluR5(A) and GAPDH (B) were presented. The dilution ratio of the antibody of mGluR5 is 1:500. In the Supplementary Figure S3 B, we have cut off the nonspecific blots above the mGluR5 and GAPDH to present the mGluR5 expression better.
